# Replicating DNA by cell factories: roles of central carbon metabolism and transcription in the control of DNA replication in microbes, and implications for understanding this process in human cells

**DOI:** 10.1186/1475-2859-12-55

**Published:** 2013-05-29

**Authors:** Sylwia Barańska, Monika Glinkowska, Anna Herman-Antosiewicz, Monika Maciąg-Dorszyńska, Dariusz Nowicki, Agnieszka Szalewska-Pałasz, Alicja Węgrzyn, Grzegorz Węgrzyn

**Affiliations:** 1 Department of Molecular Biology, University of Gdańsk, Wita Stwosza 59, Gdańsk 80-308, Poland; 2 Department of Microbiology, University of Szczecin, Felczaka 3c, Szczecin 71-412, Poland

**Keywords:** DNA replication, Central carbon metabolism, Transcription

## Abstract

Precise regulation of DNA replication is necessary to ensure the inheritance of genetic features by daughter cells after each cell division. Therefore, determining how the regulatory processes operate to control DNA replication is crucial to our understanding and application to biotechnological processes. Contrary to early concepts of DNA replication, it appears that this process is operated by large, stationary nucleoprotein complexes, called replication factories, rather than by single enzymes trafficking along template molecules. Recent discoveries indicated that in bacterial cells two processes, central carbon metabolism (CCM) and transcription, significantly and specifically influence the control of DNA replication of various replicons. The impact of these discoveries on our understanding of the regulation of DNA synthesis is discussed in this review. It appears that CCM may influence DNA replication by either action of specific metabolites or moonlighting activities of some enzymes involved in this metabolic pathway. The role of transcription in the control of DNA replication may arise from either topological changes in nucleic acids which accompany RNA synthesis or direct interactions between replication and transcription machineries. Due to intriguing similarities between some prokaryotic and eukaryotic regulatory systems, possible implications of studies on regulation of microbial DNA replication on understanding such a process occurring in human cells are discussed.

## Introduction

When considering a cell as either a subject of a basic study or a producer of recombinant proteins and natural products for biotechnological purposes, it is necessary to remember that it cannot function without proper regulation of its genome replication. If DNA replication is either severely inhibited or over-stimulated, the cell can stop to grow or even die. In some eukaryotic organisms, impairment of the negative regulation of DNA synthesis leads to cancer development. In any case, disturbance of DNA replication is a highly adverse phenomenon, irrespective of whether we treat the cell as a biological object or as a biotechnological producer. Therefore, understanding the mechanisms of regulation of this process is crucial for both basic knowledge and applied biology.

Contrary to early presentations of the processes of DNA replication and transcription occurring in prokaryotic and eukaryotic cells, in which enzymatic synthesis of nucleic acids would occur due to actions of single enzymes trafficking along template molecules somewhere in cytoplasm (in prokaryotes) or nucleoplasm (in eukaryotes), it was shown, about 20 years ago, that these processes are operated by large nucleoprotein complexes, called replication and transcription factories (for an early review, see [1]). Replication factories have been detected in human cells as ovoid bodies remaining morphologically and functionally intact despite the removal of most of the chromatin [2]. These factories were found to be attached to a cytoskeleton, with replication occurring as the template moves through them [3]. Interestingly, just one year before replication factories were discovered in human cells, stable protein complexes required for DNA replication and inherited by one of two daughter DNA copies after each replication round were reported in one of prokaryotic models used extensively in DNA replication studies – plasmids derived from bacteriophage λ [4]. The replication factory model was then supported in studies on other prokaryotic replicons, chromosomes of *Bacillus subtilis*[5], *Caulobacter crescentus*[6] and *Escherichia coli*[7].

Despite the fact that subsequent studies have provided increasing amount of information about prokaryotic and eukaryotic replication factories (see, for example [8-10]), including modifications of the original model [11,12], and addressed important problems of subcellular positioning of the replication *origin* region [13] and conflicts between replication and transcription factories (arising from the use of the same DNA template at the same time by both these cellular machineries) [14,15], our knowledge about their functions is still highly incomplete. In this review we will focus on the problem of regulation of the DNA replication process, including the control of formation and action of replication factories, which is necessary to ensure stable inheritance of the genetic material throughout potentially unlimited number of cell generations. Although the level of our understanding of such regulation is even lower than that of the structure and basic function of replication factories, recent years did bring interesting results which might shed a new light on the control of these processes. Thus, we will concentrate on recent results indicating specific influence of central carbon metabolism and the transcription processes on the control of DNA replication. We will also discuss whether principles of DNA replication regulation can be common in bacteria, bacteriophages and humans.

## Review

### DNA replication as a central biological process

From the biological point of view, it is crucial to understand which processes are common to all or most of organisms and which are specific to particular groups of organisms. Such a knowledge is not only necessary to increase our basic understanding of biological systems, but also it allows us to build models of regulatory mechanisms and to predict possible effects of various factors influencing conditions under which particular organism exists. This is especially important due to the fact that it is not possible (both physically and economically) to investigate all kinds of organisms at the level detailed enough to learn about molecular processes occurring in their cells. This is why experiments on human models are routinely preceded by studies on other selected organisms. However, it is crucial to know what information gained from experiments on various organisms can be extrapolated to human cells and what cannot.

The process of genetic material duplication can be divided into three general steps: initiation, elongation and termination [16]. Although each of these steps can potentially be regulated, it seems that vast majority of regulatory mechanisms operate at the first step – the initiation. This is reasonable as the control at the very beginning of the process may prevent energy wasting, which is usually crucial for any organism, especially under nutrient-limiting conditions. Nevertheless, it is also necessary to control the replication process at further stages, particularly if environmental or physiological conditions change suddenly, and it is crucial for the cell to react quickly. Looking at the already available literature on DNA replication, it seems that we have learned quite a lot about biochemistry of this process, including biochemical characteristics of the proteins involved in it, as well as reactions which must occur to ensure efficient DNA synthesis (for recent reviews see [17,18]). However, one may conclude that our knowledge is surprisingly poor if we focus on the regulation of DNA replication in response to various environmental or physiological conditions and factors. While some information is available about regulatory processes occurring in prokaryotic cells (see, for example, discussion in ref. [19]), it appears that molecular mechanisms of the response of DNA replication to extracellular processes and factors, as well as to physiological status of the cell, still remain to be elucidated.

The biochemical processes of DNA replication initiation involve similar steps in both prokaryotes and eukaryotes [18]. A replication initiator protein (e.g. DnaA in most bacteria) or a complex of proteins (e.g. ORC in eukaryotic cells) must bind to the region called *origin* of replication (or *ori*), determined by either specific nucleotide sequence (in bacteria and yeast) or DNA topology (in metazoans). Then, a helicase must be loaded at the region occupied by DnaA/ORC, which requires assistance of other protein(s). At this stage, there are some differences between prokaryotes and eukaryotes, as in bacteria helicase is loaded as a part of the initiation process, while in eukaryotic cells helicase is loaded earlier, in the process of licensing. Finally, helicase must be activated and DNA polymerase has to be loaded at the same site to initiate DNA synthesis. Obviously, these processes are significantly more complicated, but a general scheme can be presented as described above. For details, we refer readers to recent review articles [17,18,20-22].

Once the DNA synthesis is initiated, the second stage of the replication process, elongation, is established. Again, similar general mechanisms operate in prokaryotic and eukaryotic cells, while there are significant differences if we consider details of the reaction. Generally, activities of DNA polymerase (or polymerases), helicase, primase, ligase, and DNA topoisomerases are required [16].

The replication process finishes after duplication of the whole replicon (defined as a genome fragment being replicated from a single *origin*). Due to general basic differences in the organization of bacterial and human chromosomes (circular – in most cases - vs. linear chromosomes, respectively), the processes of DNA replication termination appear to be significantly different in prokaryotic and eukaryotic cells [16]. However, since in this review we will focus mostly on replication initiation and elongation rather than on replication termination, the latter process will not be discussed in more detail here.

As mentioned above, the process of DNA replication must be precisely regulated, particularly in response to changing environmental conditions. We have learned a lot about biochemical reactions leading to formation of the replication complexes, and further to replication factories, in both prokaryotes and eukaryotes. For this knowledge, a reader is referred to recent review articles [23,24]. Here, we focus on recently published reports, which may have influence on our understanding of global regulatory processes, controlling DNA replication in both bacteria and humans.

### The link between central carbon metabolism and DNA replication

In every heterotrophic organism, there are two basic processes ensuring that more specialized cellular reactions (like transcription of particular genes and translation of particular mRNAs by ribosomes as well as enzyme-mediated production of various compounds) can occur. These two processes are central carbon metabolism (CCM) (for a review see [25]) and DNA replication (for a review see [16]). The former one provides energy from nutrients, and supplies the precursors for biosynthetic pathways, which is absolutely necessary to all life functions of cells. The latter one, although consuming cellular energy, ensures integrity of genetic material and its inheritance by daughter cells after each cell division, providing the source of information about biological structures and functions of macromolecules.

CCM is recognized as a set of biochemical pathways devoted for transport and oxidation of main carbon sources in the cell [25]. Generally, it consists of the phosphortransferase system, glycolysis, gluconeogenesis, pentose-monophosphate bypass with Entner-Doudoroff pathway, Krebs cycle with glyoxylate bypass and the respiration chain. Biochemical reactions of these pathways ensure the optimal energy production and usage in the cell at particular environmental and physiological conditions, in order to keep homeostasis. It was known since early biochemical studies on bacterial cells that prokaryotes prefer glucose over other carbon sources, which is reflected in specific regulations of expression of genes coding for catabolic enzymes [25]. However, it is perhaps surprising that eukaryotic cells are unable to consume a readily available supply of extracellular nutrients when glucose is absent. Moreover, it was demonstrated that pools of metabolites involved in central carbon metabolism drop following glucose withdrawal [26].

The process of replicative DNA synthesis requires a large cellular machinery. In the most intensively studied model Gram-negative bacterium, *Escherichia coli*, it consists of DNA polymerase III holoenzyme (containing at least 10 subunits) and other essential proteins, including DnaB helicase and DnaG primase. Additional proteins (DnaA, DnaC) are required for DNA replication initiation at a specific genome region, called *oriC*[20]. In human cells, the DNA replicating machinery is more complex, however, general functions played by particular proteins are similar, in principle, as reviewed recently [17,18,21,22].

Although it was observed previously that regulation of prokaryotic DNA replication may depend on bacterial cell metabolism, it was generally assumed that this dependency is indirect. For example, it might result from different availability of cellular energy and/or precursors of macromolecules [27,28] or from production of specific alarmons, like cyclic AMP (cAMP) [29,30] or guanosine tetraphosphate (ppGpp) [19,31-33], in response to nutritional deprivations. However, even the action of ppGpp in the control of DNA replication may be direct, contrary to earlier assumptions that this nucleotide regulates exclusively expression of various genes. Namely, it was demonstrated that ppGpp binds DnaG primases from *B. subtilis*[34] and *E. coli*[35,36], inhibiting their enzymatic activities. Interestingly, earlier studies suggested that ppGpp inhibits DNA replication elongation in *B. subtilis* and the initiation stage in *E. coli*[31]. Although recent studies indicated that DNA replication elongation rate is decreased by high levels of ppGpp also in *E. coli*, this effect was significantly less pronounced than in *B. subtilis*[37]. Moreover, while ppGpp influences the elongation process only weakly in *E. coli* cells [37,38], it strongly inhibits DNA replication reconstructed *in vitro* from *E. coli* proteins [38]. It was hypothesized that although ppGpp inhibits activities of DnaG primases from *B. subtilis* and *E. coli* to similar level *in vitro*, this nucleotide cannot efficiently block DNA replication elongation in *E. coli* cells as it is bound mostly to RNA polymerase molecules [38]. Since *B. subtilis* RNA polymerase does not bind ppGpp, and transcription inhibition during starvation of this bacterium is caused by changes in nucleotide pools [39,40], the primase is not outcompeted by RNA polymerase for interactions with ppGpp and can be inhibited, which results in impairment of DNA replication elongation [38].

**Figure 1:**
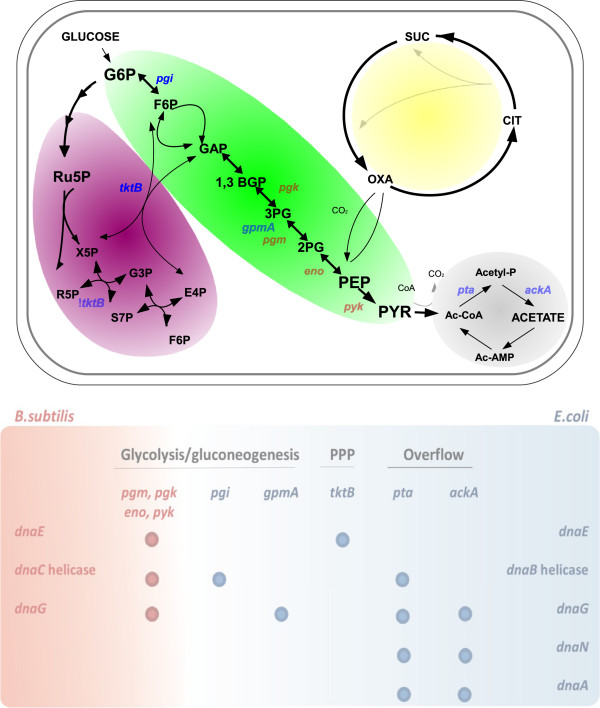
**The scheme of the central carbon metabolism (CCM, upper panel), with indicated genes coding for enzymes involved in particular reactions.** Colored backgrounds indicate glycolysis/gluconeogenesis (green), pentose phosphate pathway (violet), the overflow reactions (gray), and the Krebs cycle (yellow). Lower panel demonstrates the pattern of suppressions of effects of mutations in genes coding for replication factors in *B. subtilis* and *E. coli* by particular mutations in genes coding for CCM enzymes, involved in glycolysis/gluconeogenesis, pentose phosphate pathway (PPP) or the overflow reactions. In both panels, red color indicates specific suppressions in *B. subtilis*, and blue color indicates specific suppressions in *E. coli*. Abbreviations: **1,3-BGP**, 1,3-biphosphoglycerate; **2PG**, 2-phosphoglycerate; **3PG**, 3-phosphoglycerate; **G3P**, galactose-3-phosphate; **G6P**, glucose-6-phosphate; **F6P**, fructose-6-phosphate; **OXA**, oxaloacetate; **PBP**, fructose-1,6-biphosphate; **GAP**, glyceraldehyde 3-phosphate; **PEP**, phosphoenolpyruvate; **PYR**, pyruvate; **Ru5P**, ribulose-5-phosphate; **R5P**, ribose-5-phosphate; **S7P**, sedoheptulose-7-phosphate; **E4P**, erythrose-4-phosphate; **Ac-CoA**, acetyl coenzyme A; **Acetyl-P**, acetyl phosphate; **Ac-AMP,** acetyl-AMP; **CIT**, citrate; **SUC**, succinate; **X5P**, xylulose-5-phosphate.

Even more spectacular connection between DNA replication and metabolism has been described. It was reported recently that DNA replication may be directly linked to central carbon metabolism, particularly glycolysis, in a model Gram-positive bacterium, *Bacillus subtilis*[41]. In that study, specific suppression of conditionally-lethal (temperature-sensitive) mutations in genes coding for some replication proteins by dysfunction of certain genes coding for enzymes involved in glycolysis, was observed. Particularly, mutants in *pgm, pgk eno* and *pyk* genes reversed phenotypes of mutations in genes coding for DnaE (a DNA polymerase involved in lagging strand synthesis), DnaC (a helicase – the homologue of *E. coli* DnaB protein), and DnaG (the primase) (Figure 1). An indirect suppression mechanism (e.g. by slowing down of bacterial growth rate) was excluded, strongly suggesting the existence of a real link between glycolysis and DNA replication.

Other recent studies indicated that temperature-sensitivity of mutants in particular genes coding for replication proteins could be suppressed by deletions of certain genes coding for enzymes of the central carbon metabolism in *E. coli*[42]. The effects of *dnaA46*(ts) mutation could be suppressed by dysfunction of *pta* or *ackA*, effects of *dnaB*(ts) by dysfunction of *pgi* or *pta*, effects of *dnaE486*(ts) by dysfunction of *tktB*, effects of *dnaG*(ts) by dysfunction of *gpmA, pta* or *ackA* and effects of *dnaN159*(ts) by dysfunction of *pta* or *ackA* (Figure 1). The observed suppression effects were specific, as they could be reversed by expression of wild-type genes from a plasmid, and were not caused by a decrease in bacterial growth rate [42].

The studies discussed above [41,42] have indicated that the genetic correlation exists between central carbon metabolism and DNA replication in Gram-positive and Gram-negative model bacteria, *B. subtilis* and *E. coli*, respectively*.* This link exists at the steps of initiation and elongation of DNA replication, indicating the important global correlation between metabolic status of the cell and the events leading to cell duplication. Dysfunctions of genes coding for enzymes involved in different parts of CCM (glycolysis/gluconeogenesis, pentose phosphate pathway (PPP), and overflow reactions) caused specific suppressions of effects of mutations in replication genes [41,42]. However, it is intriguing that major effects were observed in mutants in *pgm, pgk, eno, pyk, pgi* and *gpmA* genes (whose products catalyze reactions of glycolysis/gluconeogenesis) in *B. subtilis*, while dysfunctions of *pta* and *ackA* genes (encoding enzymes of the overflow part of CCM) gave major effects in *E. coli* (Figure 1). Moreover, the suppression effects were pronounced in *B. subtilis* mostly at the elongation stage, whereas in *E. coli*, effects of mutations affecting DNA replication initiation were also suppressed. This may generally resemble ppGpp-mediated regulation of DNA replication, discussed above, where elongation and initiation stages were subjects of specific controls in *B. subtilis* and *E. coli*, respectively. This may reflect more general strategies of these two bacterial species, where regulation of DNA replication involves the elongation stage in *B. subtilis*, while it is focused on the initiation process in *E. coli*.

The above described discoveries open interesting possibilities to investigate molecular mechanisms of the CCM-dependent regulation of DNA replication in bacteria and to ask whether such a regulation is specific to bacteria or exists also in eukaryotic organisms, including humans. The latter possibility is especially intriguing in the light of the coupling of the Warburg effect to cancer cell proliferation. In fact, there are many observations positioning glycolysis as a central player in malignancy (for a recent review see [43]). In this light, it is worth to note that enzymes catalyzing the reactions leading from glucose through many intermediates are well conserved in living organisms, and the reactions and their products are very similar. Moreover, in all organisms, doubling of the genetic material has to be strictly correlated with the overall status of the cell physiology. In eukaryotic cells, there are several precisely controlled check-points in the cell cycle, ensuring that the next step could start only upon completion of all requirements of the previous one (see, for example, [44]). However, the general and global regulation involving the interplay between the cell metabolic status and DNA replication could be an important mechanism to correlate these processes, to ensure the proper and correct DNA synthesis and genome stability.

The stability of the genome can be affected by the replication impairment, due to either errors in DNA synthesis or stalled and collapsed replication forks. In bacteria, mutations occurring as an effect of such events could lead to decreased viability and survival. In eukaryotic organisms, especially in metazoans, such mutations rarely cause the death directly, but they could lead to accumulation of alterations in the genome, resulting in life-threatening illnesses such as cancer. It is, thus, intriguing that mutations in genes coding for CCM enzymes influence not only frequency and efficiency of DNA synthesis, but also fidelity of DNA replication in *E. coli*[45]. Specifically, the mutator phenotypes (expressed as increased frequencies of mutations appearing during the DNA replication process) of *E. coli dnaQ49* and *dnaX36* mutants were either suppressed or enhanced by dysfunctions of *zwf, pta, ackA, acnB* and *icdA* genes. Overexpression of appropriate wild-type CCM genes in double mutants (containing one mutation in either *dnaQ49* or *dnaX36* and another in one of CCM genes) resulted in reversions to the initial mutator phenotypes, indicating that the effects were specific. These suppression and enhancement effects were not caused by changes in bacterial growth rates [45]. On might speculate that if such a mechanism operates also in eukaryotic cells, metabolic disturbances could lead to increased mutagenesis and increased risk of cancer development.

On the other hand, one can imagine that the effect of metabolic mutations on DNA replication fidelity could give bacteria the advantage in natural environment. In fact, in the prolonged stationary phase culture of *Streptococcus* sp., the bacteria overtaking the population were isolated and identified as mutants in genes coding for enzymes of the overflow pathway [46]. Nevertheless, irrespective of specific biological effects in various organisms (from bacteria to humans) these results corroborate the conclusion that CCM reactions may influence genome stability and that changes in particular metabolic reactions may result in increased mutagenicity.

What are putative mechanisms of the interrelations between CCM and DNA replication regulation? One possibility is that metabolites may play the role of the link between DNA replication and CCM pathways. Deficiency of CCM enzymes may lead to accumulation of certain metabolites that otherwise, in wild-type cells, would be substrates for subsequent reactions (e.g. accumulation of acetyl-phosphate in cells lacking *ackA*, citrate in the *acnB* mutant etc.). This, besides metabolic disturbance and possible slowing-down of growth, may have additional effects. Namely, some of the metabolites may serve as signal molecules and/or a source of active groups for modification of other proteins. In fact, one of the metabolic intermediates, acetyl phosphate, was reported to play a role in intracellular signaling and protein stability [47]. The metabolites could plausibly serve as low-molecular-weight signals, to carry information about the metabolic status of the cell and causing the necessary adjustments in DNA replication and subsequent cell division. Replication factories, as large complexes with many intermolecular connections, could be particularly suitable candidates to sense such signals and to convert the information into specific changes in the factory structure and function, leading to modulation of the replication control.

The cells with replication defects usually present phenotypic features including slow growth and problems with cell division. In bacteria, this may result in filamentous cells and alteration in nucleoid position. Recent microscopic analysis of the replication mutants revealed that this defect could also be suppressed by CCM mutations [48]. It was demonstrated that deletion of the *pta* and *ackA* CCM genes significantly reduced the cell length in the replication mutants *dnaA46*, *dnaB8*, *dnaE486*, *dnaG(ts)* and *dnaN159*. Such an effect was also found in the *tktB dnaE486* double mutant. Moreover, CCM enzyme dysfunction restored the nucleoid shape and position in double mutants [48]. These observations suggest again that CCM-involved enzymes can have a global effect on cell physiology, which is mediated by modulation of the DNA synthesis process controlled by replication factories.

In addition to the existence of the putative metabolite-based signal transduction between CCM and DNA replication, there is another possibility to transfer the information about the metabolic status to the replication machinery. Some enzymes were identified as multifunctional proteins in which two functions are found in a single polypeptide chain, the phenomenon referred to as moonlighting enzymes [49]. Metabolic proteins can play such a role; in fact their ability to modify other proteins by glycosylation or methylation has already been shown in a few cases [49]. The idea about this possibility comes from the option of trigger enzymes – proteins that can control gene expression in addition to their catalytic activity. The moonlighting features as transcription regulation of glycolytic enzymes were shown in eukaryotic cells [50]. In *E. coli*, one of enzymes involved in the glycolysis pathway, enolase, can be a part of RNA degradosome [51]. Therefore, there are plenty potential possibilities of modifications of replication proteins’ functions in accordance with metabolic activity of the cell, which is correlated with nutrient and energy abundance and general status of the organism. Again, replication factories are structures significantly more likely to adopt moonlighting enzymes and to sense specific information they provide than any single protein.

In summary, it is clear that cell metabolism directly affects regulation of DNA synthesis in as different organisms as bacteria and humans. Undoubtedly, elucidating the principles of regulatory mechanisms linking DNA replication to CCM in prokaryotic and eukaryotic cells will be a very important step in our way to understand the network of control processes influencing cell functions. Nevertheless, irrespective of the molecular nature of the link and the signal, replication factories are the best candidates for sensors of such signals and for structures that may convert the signals and respond appropriately to modulate the DNA synthesis process.

### The role of transcription in regulation of DNA replication initiation

Although properties of proteins involved in DNA replication and biochemical reactions occurring during this process are relatively well investigated, molecular mechanisms of the replication initiation regulation are still largely unknown. It was demonstrated that in eukaryotic (including human) cells, the replication initiation frequency is modulated by the process of transcription [18,22,52]. Contrary to bacterial replicons, in which DNA helicase is loaded at the *ori* region in an active form, eukaryotic helicase requires activation to start DNA unwinding, which is necessary to initiate DNA synthesis by DNA polymerase [18,20,21]. Nevertheless, it is intriguing that the process of replication initiation regulation in eukaryotes resembles that found in bacteriophage λ more than that in bacterial chromosome [53]. In this light, it is worth to note that while molecular mechanisms of DNA replication initiation regulation are poorly defined in human cells, there is quite a large background of such a regulation in λ-derived replicons. Therefore, we will summarize this knowledge briefly below.

The *origin* of bacteriophage λ DNA replication, called *ori*λ, is located in the middle of the *O* gene (for reviews on principles of phage lambda DNA replication see [19,53,54]. The *O* gene codes for the replication initiator protein, which binds to the replication origin, forming the nucleoprotein structure called ‘O-some’. The second λ replication protein, the *P* gene product, is involved in delivery of the host (*E. coli*)-encoded DNA helicase, the DnaB protein, to the O-some. Thus, the *ori*-O-P-DnaB structure, called ‘preprimosome’ is formed. This structure (analogously to eukaryotic systems – compare [53] and [18]) is stable but inactive in promoting DNA replication due to strong interactions between P and DnaB proteins, which prevents the helicase activity of the latter component. Therefore, remodeling of the preprimosome is necessary, which is performed by the action of heat shock proteins (molecular chaperones): DnaK, DnaJ and GrpE.

Despite the fact that the remodeling of the preprimosome is necessary to liberate DnaB from P-mediated inhibition, the P protein seems to be still present in the complex. DnaK also remains bound to the *ori*-O-P-DnaB complex [8]. Importantly, heat shock protein-dependent preprimosome remodeling is coupled with transcriptional activation of *ori*, a process of transcription proceeding in the replication *origin* region. Transcriptional activation of the *origin* is necessary for efficient initiation of λ DNA replication *in vivo* even if all replication proteins are provided, which resembles the process described in eukaryotic cells (for a review, see [53]). It was suggested that changes in DNA topology, caused by movement of RNA polymerase during transcription, may play a crucial role in stimulation of the replication initiation [55]. However, results of recent studies indicated that RNA polymerase directly interacts with the O replication initiator protein, which shed a new light on transcriptional activation of the *origin*[56]. One might suppose that a large nucleoprotein complex, which includes RNA polymerase, operates during the λ DNA replication initiation. In eukaryotic cells, such a complex is definitely larger and more compound than that of bacteriophage λ, but we believe it would be intriguing to investigate whether it may contain factors which were not previously supposed to participate in its structure.

Once the helicase is de-repressed (or activated, depending on whether we are describing the λ replicon or another kind of replicon, and what nomenclature is used by particular researchers), the final step of the initiation of λ DNA replication can occur. It consists of binding of DNA polymerase III holoenzyme and accessory replication proteins (primase, DNA gyrase, SSB and other), encoded by the host, to the *ori* region, and starting DNA synthesis. Obviously, formation of replication factories includes these reactions.

From the overview provided above, it appears that transcription may be the crucial regulatory process in the control of frequency of λ DNA replication initiation. It is important to remember that the *p*_R_ promoter is a natural start site for transcription that produces mRNA for synthesis of λ replication proteins (O and P) and acts to stimulate *ori*-initiated replication.

Contrary to early assumptions that λ replication complex is disassembled after initiation of a replication round, it was demonstrated that this structure is stable, and can survive in a potentially active form for at least several cell generations [4,55,57]. Moreover, this structure, which includes O, P, DnaB and DnaK proteins [8], is randomly inherited by one of the two daughter DNA copies after each round of replication. In fact, this phenomenon is a more general process rather than restricted only to λ, as eukaryotic *origin* recognition complex, ORC, also remains bound to DNA after the start of replication (discussed in [53]).

Demonstration of stability and inheritance of the λ replication complex excluded a previously assumed crucial role of the O protein instability in the regulation of λ DNA replication. In fact, several lines of evidence led to conclusion that firing of *ori*λ depends on transcriptional activation of this region [55]. If so, factors that influence activity of the *p*_R_ promoter should play crucial roles in the regulation of λ DNA replication. In this light, it is of special interest to understand molecular details of this regulation. Because of a general similarity between the λ system and that operating in human cells (see above), studies on transcription regulation in the region neighboring the λ DNA replication start point may have implications for understanding of the replication initiation control in human cells. It is worth mentioning that in human cells, like in other metazoan cells, there are no specific DNA sequences determining the *origin* sites. Nevertheless, it was possible to indicate *origin* regions at which DNA replication starts specifically [52]. It is assumed that special DNA topology or structure, rather than a nucleotide sequence, may be recognized as a region to which ORC binds. Interestingly, a recent report [58] indicated that *origin*s of DNA replication in human cells are preferentially located at the 5′-ends of highly expressed genes. This is another strong suggestion that DNA replication initiation is regulated, or at least significantly modulated, by the transcription process in human cells. Therefore, in both these forms of life, λ and humans, transcription seems to play a crucial role in the regulation of DNA replication initiation [18,19,53,54].

As mentioned above, a new light on the regulation of λ DNA replication process was shed by demonstration of direct interactions between RNA polymerase and the replication initiator protein, the lambda *O* gene product [56]. Moreover, another work [59] confirmed previous hypothesis, based on genetic experiments [60], that RNA polymerase directly interacts with DnaA protein, which in the λ system acts as an activator of the promoter that serves as a start point of transcription proceeding in the *ori* region. Therefore, we suggest that a large nucleoprotein structure, significantly larger than supposed previously, is formed at the *ori*λ region. The RNA polymerase-λO protein interactions result in considerable changes in binding of the replication initiator protein to the *ori*λ region [56]. This must influence the replication initiation process.

**Figure 2:**
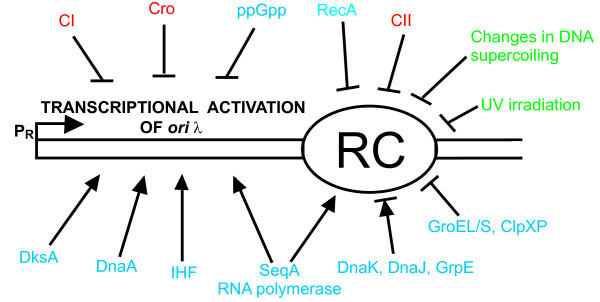
**Factors and processes influencing transcriptional activation of the *origin *and replication complex (RC) function in bacteriophage λ DNA replication.** Positive regulations are marked by arrows, and negative regulations are symbolized by blunt-ended lines. Host-encoded and host-produced factors are marked in blue, bacteriophage-encoded factors – in red, and physical factors – in green. For detailed review on the transactions depicted in this scheme, see ref. [64].

The second recent breakthrough in studies on λ DNA replication was discovery that very few differences in replication *origin* regions of various lambdoid phages may result in dramatic changes in the control of the replication initiation. It was shown that as little difference as a single amino acid substitution in the O protein, and a single amino acid substitution in the P protein, may cause independence of lambdoid plasmid replication on the host-encoded DnaA protein, which is a stimulator of the transcriptional activation of the *origin*[61,62]. Moreover, some lambdoid phages contain 6 sequences which bind the O protein at *ori*, rather than 4 (like in λ) [61]. Therefore, it must be a delicate balance between various interactions of particular players in the process of the control of λ DNA replication. It is worth reminding that the promoter which is responsible for initiation of transcription leading to transcriptional activation of the *origin*, the *p*_R_ promoter, was found to be regulated by several factors. Beside the already mentioned DnaA protein, which stimulates the activity of *p*_R_, other proteins, like cI, Cro (negative regulators), SeqA, DksA (positive regulators) or nucleotides, like pppGpp and ppGpp (negative regulators), are important modulators of the *p*_R_ activity [63] (Figure 2). In this light, it is tempting to speculate that an interplay between two kinds of microbial cell factories, replication and transcription factories, occurs during the replication initiation at *ori*λ, and that this interplay may be crucial for the control of this process. Therefore, it appears that functions of replication and transcription factories are coupled not only antagonistically (or negatively), due to conflicting processes that occur competitively on the same template, but also synergistically (or positively), during DNA replication initiation. As shown in Figure 2, there are several factors (RNA polymerase, DnaA, DksA, IHF, SeqA, CI, Cro, ppGpp) that affect efficiency of transcriptional activation of *ori*λ (mainly by modulating the *p*_R_ promoter activity), and several factors and processes (DnaK, DnaJ, GrpE, SeqA, RNA polymerase, GroEL, GroES, ClpXP, CII, RecA, UV irradiation, changes in DNA supercoiling) that influence formation and stability of the replication complex. Some factors, like RNA polymerase and SeqA, participate in both processes, which may strengthen the hypothesis about cooperation between replication and transcription factories at the *ori*λ region during initiation of phage λ DNA replication.

When comparing DNA replication in λ and human cells, as mentioned above, one should note that vast majority of information on eukaryotic DNA replication comes from studies on yeast or frog embryos [22]. However, these models differ considerably from human cells regarding the *origin* structure and function [52]. As mentioned in one of previous paragraphs, recent results indicated that human *origins* of replication are preferentially located at the regions of promoters of highly active genes [58]. Moreover, locations of these *origins* correlated with locations of RNA polymerase II binding sites [58]. Thus, we suspect that employing the knowledge gained from studies on the λ model should allow to obtain important results, facilitating formulation of hypotheses on the mechanisms of regulation of human DNA replication initiation.

### Combination of regulatory systems in a single organelle

There is a specific organelle, in which regulation of DNA replication is strictly connected with both processes described in this article. CCM and transcriptional activation influence not only the control of nuclear DNA replication, but also modulate the mitochondrial DNA (mtDNA) synthesis process. In the light of possible common mechanisms operating in the regulatory processes that occur in both prokaryotic and eukaryotic cells, discussed in previous subsections, it is important to remember that according to the generally accepted evolutionary model, mitochondria (together with chloroplasts) can be considered as relicts of ancient bacterial endosymbionts living inside eukaryotic cells [65].

**Figure 3:**
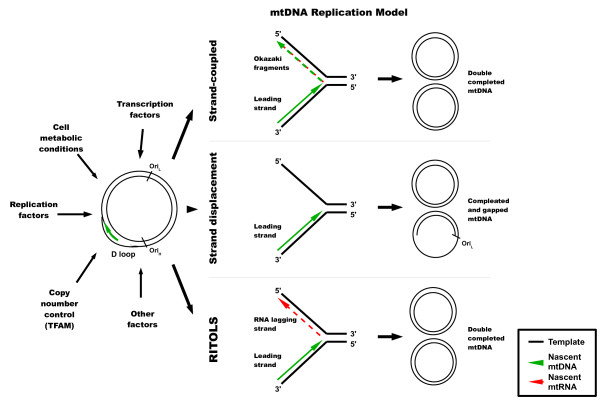
**Models of mtDNA replication.** Three major models are presented (for detailed review, see ref. [66]) with nascent DNA and RNA strands depicted by green and red arrows, respectively. A simplified model for mtDNA, with Ori_L_, Ori_H_ and D-loop regions, is shown at the left side of the scheme, together with factors and processes influencing mtDNA replication. Final products of the reactions are shown at the right side of the scheme.

There are three main models proposed for mtDNA replication, each requiring transcription and/or transcription factors (for a review see ref. [66]). These models, together with factors and processes involved in mtDNA replication and its regulation, are presented schematically in Figure 3.

Replication of mtDNA does not coincide with the cell cycle and occurs independently of nuclear DNA replication [67]. However, there is evidence that the initiation of the heavy-strand mtDNA replication from the H-strand origin (Ori_H_) requires transcription initiated at the light strand promoter (LSP) and is carried out by nuclear-coded factors [68,69]. This transcription is assisted by the mitochondrial transcription factor A (TFAM) which, thus, may regulate mtDNA copy number [70,71].

Mitochondria are responsible for supplying cellular energy, and they regulate cellular metabolism. Most diseases associated or coupled with a decrease in mtDNA copy number (mtDNA depletion) have been linked to mutations in various nuclear genes that code for either mitochondrial DNA polymerase (*POLG*) or enzymes involved in mitochondrial nucleotide metabolism, such as *TK2, DGUOK, SOCLA2* and *PEO1* (for a review see ref. [72]). This confirms the relationship between DNA replication and cellular metabolism, as in a wild-type organism the efficiency of production of nucleotides depends on the metabolic status of the cell. Moreover, it was demonstrated that high concentrations of glucose increased the activity of the rat *Tfam* promoter, indicating that the mtTFA gene expression (and hence, mtDNA replication) is regulated by exogenous or metabolic stimuli [73,74].

Apart from the mechanism by which a higher frequency of TFAM binding at LSP increases the efficiency of transcription-mediated priming of replication, there is another process significantly influencing mtDNA metabolism. Namely, binding of TFAM to mitochondrial genome may reduce the rate of DNA turnover, which stabilizes steady-state levels of mtDNA [75]. Thus, both mechanisms, in mutually coupled manner, ensure the proper amount of mtDNA in cells.

## Conclusions

Results of recent studies indicated that two processes, central carbon metabolism (CCM) and transcription, significantly influence the control of DNA replication of microbial replicons. Microbial replication factories are the most likely candidates for sensors of signals produced either during CCM (i.e. specific metabolites) or independently by enzymes involved in this metabolic pathway (i.e. moonlighting enzymes). It is also likely that two kinds of cellular factories, replication and transcription factories, cooperate in the process of replication initiation regulation. Thus, one might also speculate that a specific metabolic signal can be transmitted to the replication factory through the transcription factory, especially since some moonlighting enzymes were found to be involved in the regulation of RNA synthesis and degradation. Finally, due to general similarities of the metabolic and regulatory processes occurring in prokaryotic and eukaryotic cells (including human cells), determining the principles of the mechanisms controlling DNA replication in the former ones may have implications for understanding such mechanisms operating in latter ones.

## Competing interests

The authors declare no competing interests.

## Authors’ contributions

SB, MG, AH-A, MM-D, DN, AS-P, AW and GW contributed to analyzing published data, discussing the previously published results and preparing the manuscript. All authors read and approved the final manuscript.
